# Prophylactic Treatment with Vitamins C and B2 for Methotrexate-Induced Gastrointestinal Mucositis

**DOI:** 10.3390/biom11010034

**Published:** 2020-12-29

**Authors:** Ana Rita da Silva Ferreira, Hannah R. Wardill, Rick Havinga, Wim J. E. Tissing, Hermie J. M. Harmsen

**Affiliations:** 1 Department of Medical Microbiology, University of Groningen, University Medical Center Groningen, Hanzeplein 1 EB80, 9713 GZ Groningen, The Netherlands; a.r.da.silva.ferreira@umcg.nl; 2 Department of Pediatrics, University of Groningen, University Medical Center Groningen, Hanzeplein 1 EB80, 9713 GZ Groningen, The Netherlands; hannah.wardill@adelaide.edu.au (H.R.W.); h.havinga@umcg.nl (R.H.); w.j.e.tissing@umcg.nl (W.J.E.T.); 3 Adelaide Medical School, University of Adelaide, Adelaide, SA 5005, Australia; 4 Princess Maxima for Pediatric Oncology, 3584 CS Utrecht, The Netherlands

**Keywords:** chemotherapy-induced mucositis, methotrexate, gut microbiota, anaerobic bacteria, vitamin C, vitamin B2

## Abstract

Mucositis is a common side-effect of chemotherapy treatment, inducing alterations in the composition of the gut microbiota. Redox active compounds, such as vitamins B2 and C, have been shown to reduce inflammation and enhance the growth of anaerobic bacteria in the gut. We therefore aimed to (1) validate the ability of these compounds to promote bacterial cell growth in vitro, and (2) determine their prophylactic efficacy in a rat model of methotrexate (MTX)-induced mucositis. Bacterial growth curves were performed to assess the growth kinetics of bacteria exposed to Vitamins C and B2 (0.5 mM). Male wistar rats (150–200 g) received vitamins B2 (12 mg/day) and C (50 mg/day) via daily oral gavage (from day −1 to day 10). MTX (45 mg/Kg) was administrated via I.V. injection (N = 4–8/group) on day 0. Body weight, water/food consumption and diarrhea were assessed daily. Blood and faecal samples were collected longitudinally to assess citrulline levels (mucositis biomarker) and gut microbiota composition. Vitamins C/B2 enhanced the in vitro growth of anaerobic bacteria *Blautia coccoides* and *Roseburia intestinalis*. Contrarily to vitamin B2, in vivo administration of Vitamin C significantly attenuated clinical symptoms of mucositis. Despite their influence on the composition of the gut microbiota, both vitamins did not modulate the course of MTX-induced mucositis, as accessed by plasma citrulline. Vitamins B2 and C enhanced anaerobic bacterial growth in vitro, however their ability to mitigate MTX-induced mucositis was limited.

## 1. Introduction

Gastrointestinal mucositis is one of the most common and clinically significant side effects of anticancer therapy [1,2]. Patients undergoing chemotherapy present with a spectrum of complications to mucositis, including nausea/vomiting, diarrhea, abdominal pain, infection and malnutrition. These symptoms require significant supportive care interventions, leading to longer hospitalization, thus impacting the patient’s quality of life. In severe cases, chemotherapy dose reductions or complete cessation of treatment are required, compromising the survival of patients [1].

Gastrointestinal mucositis is initiated by direct cytotoxic injury to highly proliferative intestinal stem cells, exacerbated by a secondary wave of indirect injury mediated by innate immune activation, aberrant inflammatory signaling and microbial disruption [3]. While a large number of studies have demonstrated benefits of targeting each of these specific mechanisms, few of these have translated clinically, presumably due to the narrow spectrum of disease mechanisms being targeted. In contrast to existing approaches, vitamins present as an easy and encouraging prophylactic approach due to their ability to simultaneously modulate multiple aspects of mucositis pathogenesis, including oxidative stress, inflammation and microbial disruption [4,5,6]. 

Vitamins C and B2 are essential nutrients for a normal metabolism. The recommended daily dose of vitamin C for an adult male is 90mg, whereas vitamin B2 is 1.3 mg [7]. Vitamins C and B2 are of particular interest due to the increased evidence supportingtheir immunomodulatory properties and the impact on the microbiota, particularly under oxidative stress [8,9,10,11]. In animal models of colitis, these vitamins were shown not only to ameliorate symptoms but also to enhance the growth of commensal bacteria in the gut such as *Lactobacillus plantarum* [12,13,14]. In the setting of mucositis, multivitamin use prior to therapy has been reported as a modulator of mucositis outcome, providing protection to stem cell transplantation recipients [15]. Similarly, vitamin C has been shown to attenuate 5-fluorouracil-induced gastrointestinal mucositis by inhibiting the activation of the NF-κB pathway and by reducing lipid peroxidation and myeloperoxidase (MPO) [8].

Vitamin B2, or riboflavin, has been most extensively characterized for its impact on the microbiota. While not directly assessed in the setting of mucositis, *Faecalibacterium prausnitzii*—a key butyrate producer with reduced abundance following chemotherapy—is particularly dependent on vitamin B2 as a redox mediator for extracellular electron transfer, thus reducing oxidative stress which highlights the potential of vitamin B2 in mucositis prevention [9]. The current study therefore aimed to (1) evaluate the impact of vitamin C and B2 on the growth properties in vitro of bacterial strains that are frequently associated with dysbiosis during chemotherapy-induced mucositis, and (2) evaluate their prophylactic efficacy in a validated preclinical model of methotrexate-induced gastrointestinal mucositis.

## 2. Materials and Methods

### 2.1. Bacterial Strains and Culturing Conditions

*Blautia coccoides*, *Roseburia intestinalis*, *Enterococcus faecalis* and *Escherichia coli* were isolated from faecal samples collected from Wistar rats and identified by Sanger sequencing of the 16S rRNA-gene [16,17]. Bacteria were isolated from rat faecal samples at 37 °C on yeast extract, casitone, fatty acid and glucose (YCFAG) agar under anaerobic conditions [18]. The strains were grown in YCFAG broth to an optical density at 600 nm (OD_600_) of 0.8. Bacteria were stored in YCFAG containing 20% glycerol at −80 °C prior to use in the assays.

### 2.2. Bacterial Growth Experiments

For growth experiments, a bacterial overnight culture was added to 100 µL of YCFAG supplemented with 0.5 mM of vitamin B2 or vitamin C, or both, to an OD_600_ of 0.05. To create a semi-anaerobic environment, cell suspensions were plated in 96 well plates and properly sealed with a plate sealer inside the anaerobic cabinet (nitrogen (90%), hydrogen (5%) and CO_s_ (5%)). The sealer tape allowed penetration of low amounts of oxygen. The plate was transferred to a Biotek Synergy 2 system in which growth curves were measured under static conditions at 37 °C and an OD of 600 nm measured every 4 h for 48 h. Each growth curve represented the average for three biological replicates.

### 2.3. Materials

Methotrexate (MTX) was used as a representative anticancer therapy. MTX (50 mg/kg) was obtained from Pharmachemie Holding B.V. (Haarlem, The Netherlands). The dose of MTX (45 mg/kg) administrated to the animals was based on previous studies performed in our lab [19]. Vitamin C (L-ascorbic acid; A0278-25G) and Vitamin B2 (Riboflavin; R4500-25G) were obtained from Sigma-Aldrich (Schnelldorf, Germany).

### 2.4. Animals and Ethics

This study was performed according the ARRIVE guidelines for the accurate and reproducible reporting of animal research.

### 2.5. Ethical Statement and Husbandry Specifications

The experimental protocol was approved under number 15338-01-004 by the Ethics Committee for Animal Experiments, Faculty of Medical Sciences, University of Groningen, The Netherlands. Male Wistar outbred rats (6–8 weeks old, 100–120 g) were obtained from Harlan (Horst, The Netherlands). Animals were individually housed in conventional, plexiglass cages (42.5 × 26.6 × 18.5 cm) at the Central Animal Facilities, University Medical Center Groningen. Temperature was controlled (21 ± 1 °C) with a relative humidity of 55 ± 10% in 12:12-h light-dark cycle (lights on 7 a.m.–7 p.m.). Autoclaved AIN93 G rodent diet was obtained from Research Diet Services (Wijk bij Duurstede, The Netherlands) (6.3 mg vitamin B2 and no vitamin C addition) and sterile water were available ad libitum. Sawdust bedding was provided in all cases, as well as toilet rolls for enrichment. All cages were randomly arranged across racks to prevent potential biases.

### 2.6. Experimental Procedures

**Figure 1:**
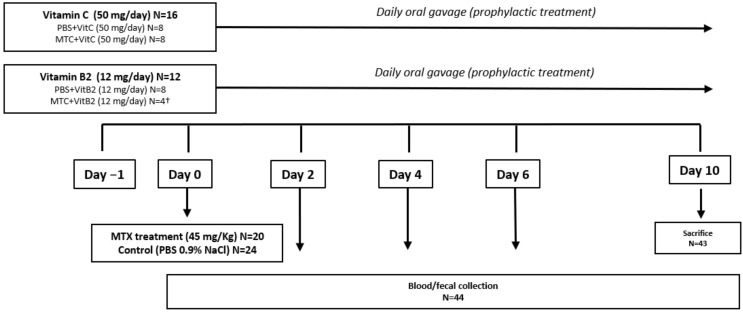
The experimental approach. 8-week-old Wistar male rats were orally gavaged with 50 mg/day vitamin C (N = 16) or 12 mg/day Vitamin B2 (N = 12) every day starting one day before methotrexate (MTX) injection. At days 0, 2, 4, 6 and 10, blood and fecal samples were collected and stored for analysis. At day 10, rats (N = 43) were sacrificed. † At day 3, one animal from MTX+VitB2 group was sacrificed due to severe symptoms. Blood and fecal samples from this animal were collected and results included at day 4.

Forty-four rats were injected intravenously at day 0 with MTX (45 mg/Kg; N = 20) or with saline solution (0.9%, N = 24), under 3% isoflurane anesthesia as previously reported [20,21,22]. Assignment of rats was performed randomly prior to any treatment with mild adjustments made to ensure comparable baseline body weight. Rats were treated with either sterile water, vitamin C (50 mg/day) or vitamin B2 (12 mg/day) via oral gavage every day, starting one day before MTX injection (Figure 1). At day 10, all animals were sacrificed via isoflurane anesthesia and cervical dislocation. The primary outcome of the study was gastrointestinal toxicity, defined by plasma citrulline levels. Secondary outcomes included food intake, body weight, water intake and presence of diarrhea (present as watery diarrhea or completely absent) were recorded daily. Contrarily vitamin C, in which optimal dose was calculated according to previous studies, no similar studies with vitamin B2 in rats have been done [8]. This has led us to first test different doses of vitamin B2: 8, 24 and 48 mg/kg. For each of these doses, 4 animals were included in the group (data not shown). Since none of the doses resulted in gut toxicity, we included the results obtained for 48 mg/kg (12 mg/day). This optimization, together with ethical issues associated with the number of animals used, resulted in a lower number of animals in the MTX+VitB2 group.

### 2.7. Plasma Citrulline Levels Determination

The primary outcome of this study was plasma citrulline concentration. Plasma citrulline is an indicator of small enterocyte mass and a validated biomarker of mucosal injury [22]. Repeated blood samples (75 μL) were collected from the tail vein into EDTA-treated hematocrit capillary tubes. Citrulline was determined in 30 μL of plasma (isolated from whole blood via centrifugation at 4000× *g* for 10 min) using automated ion-exchange column chromatography as previously described [22,23].

### 2.8. Microbiome Analysis

#### 2.8.1. DNA Extraction

DNA was extracted from faecal samples using the double bead-beater procedure adapted from Yu and Morrison (2004) and QIAamp DNA Stool Minikit guidelines (Qiagen, Hilden, Germany). Briefly, 0.25 g stool was homogenized in 1 mL lysis buffer, containing 500 mM NaCl, 50 mM Tris-HCl (pH 8), 50 mM EDTA, 4% SDS, with 0.5 g 0.1 mm zirconia beads and four 3 mm glass beads. The faecal homogenate was then heated at 95 °C for 15 min, mixing every 5 min, before being centrifuged at 4 °C for 5 min (12,000–14,000 rpm). The supernatant was collected and set aside, and the procedure repeated for the pelleted stool. Nucleic acids were then purified using selective precipitation of cell debris with 260 μL 10 M ammonium acetate. Samples were incubated on ice for 5 min, before being centrifuged at 4 °C for 10 min (12,000–14,000 rpm). The supernatant was collected and the pellet discarded, and the procedure repeated. One volume of ice-cold isopropanol was added to the supernatant and the samples were incubated on ice for 30 min. Nucleic acids were pelleted by centrifugation (15 min, room temperature), washed with 70% ethanol and allowed to dry for 5 min at room temperature. Nucleic acids were dissolved in AE buffer (10 mM Tris-Cl; 0.5 mM EDTA; pH 9.0) and stored at 4 °C overnight. The following day, samples were incubated with 2 μL (10 mg/mL) DNase-free RNase and incubated at 37 °C for 15 min. Proteinase K and buffer AL (Qiagen Minikit products) were added and samples were incubated at 70 °C for 10 min. DNA was then transferred to the QIAmp column and wash steps were performed as per the manufacturer’s guidelines. The final DNA was eluted in AE buffer and yield/quality was quantified using a NanoDrop UV Visible Spectrophotometer (Thermo Fisher Scientific, Waltham, MA, USA).

#### 2.8.2. PCR Amplification of V3 and V4 Region of the 16S rRNA Gene

Samples were diluted to 100 ng/μL for all PCR amplification procedures. Each PCR reaction contained 1 μL of 10 μM 341f forward primer, 25 μL Phire HS II Master Mix, 22 μL DNase free water, 1 μL of 10 μM 806r barcoded reverse primer and 1 μL DNA template (100 ng/μL). The sequence of the primers used in this study is listed in Supplementary Table S1. These were denatured at 98 °C for 30 s, and amplified over 31 cycles of 98 °C for 5 s, 50 °C for 5 s, and 72 °C for 10 s. Samples were held at 72 °C for 1 min and kept at 4 °C until collection. Amplification was confirmed using gel electrophoresis.

#### 2.8.3. PCR Purification, Library Preparation and Illumina Sequencing

Size selection and fragment removal was achieved using AMPure XP beads which were added at a 1:2 ratio to the PCR product. DNA was eluted in 52.5 μL 10 mM Tris HCL (pH 8.5) and quantified using the Qubit quantification kit (Thermo Fisher Scientific, Waltham, MA, USA) and the Qubit® 2.0 Fluorometer (Thermo Fisher Scientific, Waltham, MA, USA). DNA was diluted to a working concentration of 2 mM and 5 μL of each sample was pooled to form a single library and stored at −20 °C until sequencing. Sequencing was performed using the MiSeq Benchtop Next Generation Sequencer (Illumina, San Diego, CA, USA) following the manufacturer’s guidelines.

#### 2.8.4. Statistical Analysis and Bioinformatics

All data sets were assessed for normality using D’Agostino and Pearson and Kolmogorov-Smirnov analyses using GraphPad Prism version 8. If normality was confirmed, data were analyzed using a mixed-effect model with Tukey’s post-hoc testing for multiple comparisons. In cases where normality was not achieved, a non-parametric Friedman’s test with Dunn’s post-hoc testing for multiple comparisons was used to determine significance. 16S rRNA sequencing analysis was performed using Qiagen CLC genomics workbench, kindly provided by Dr. Hannah Wardill. The calculation of alpha diversity (Shannon index) and OTUs were performed using CLC Microbial Genomic Module package on the taxonomic level of genera. β-diversity was presented using principal coordinates analysis (PCoA). PERMANOVA analysis (based on Bray–Curtis dissimilarity) was used to measure effect size and significance on β-diversity for grouping variables. Bonferroni-corrected *p*-values (which correct for multiple testing) were presented in this study. In all cases, *p* < 0.05 was considered statistically significant. Statistical analyses for all data sets are outlined in figure legends.

## 3. Results

### 3.1. In Vitro Findings

#### Vitamins C and B2 Enhance Growth of Anaerobic Bacteria under Oxidative Stress

**Figure 2:**
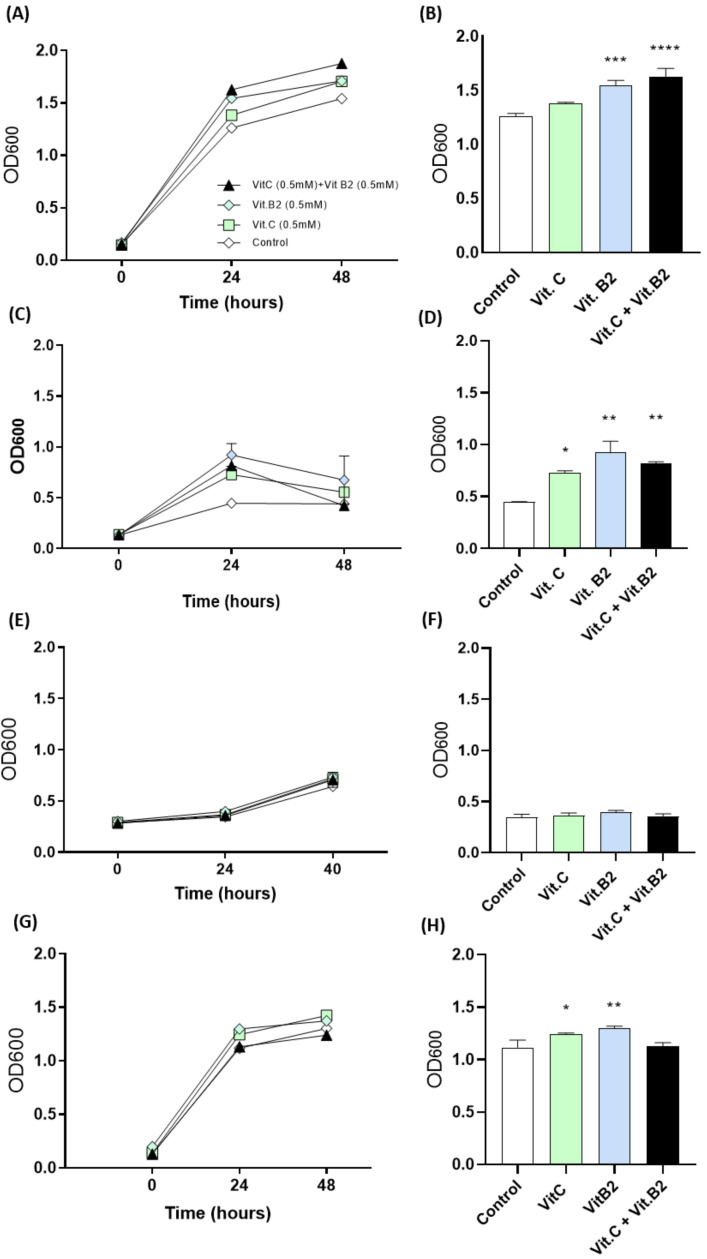
The effect of vitamins B2 and C on the growth of *B. coccoides* (**A**,**B**), *R. intestinalis* (**C**,**D**), *E. coli* (**E**,**F**) and *E. faecalis* (**G**,**H**). Bacteria stimulated with 5 mM of vitamin C and vitamin B2, alone or in combination, were grown under oxidative conditions for 48 h. Optical density measurements (600 nm) at 0, 24 and 48 h of incubation (**A**,**C**,**E**,**F**) and changes between groups were represented separately after 48 h of incubation (**B**,**D**,**F**,**H**). All data are presented as mean ± SEM. * *(p < 0.05)*, ** (*p* < 0.01), *** (*p* < 0.001) and **** (*p* < 0.0001) represent the significant changes between non-supplemented bacteria (control) and supplemented bacteria (N = 3).

We selected *B. coccoides* and *R. intestinalis* (as members of *Lachnospiraceae*) due to their beneficial role in the gut, such as the production of SCFAs [24,25,26]. Since *Enterobacteriaceae* and enterococci are often associated to a dysbiotic gut, we selected *E. faecalis* and *E. coli* as reference members [21,27]. Under mild oxidative conditions, supplementation of *B. coccoides*, *R. intestinalis* and *E. faecalis* with 0.5 mM of vitamin C and 0.5 mM of vitamin B2, alone or in combination, enhanced their growth when compared to the control (Figure 2). Supplementation of *B. coccoides* with either vitamin B2 alone (*p* = 0.0004) or the combination of both vitamins (*p* < 0.0001) resulted in significant growth after 24 h of incubation, when compared to the control group (Figure 2A,B). *R. intestinalis* growth was less significant when compared to *B. coccoides* (Figure 2C). Nevertheless, supplementation of this bacterium with vitamin C (*p* = 0.0226), vitamin B2 (*p* = 0.0011) or both (*p* = 0.0051) resulted in significant increase at 24 h (Figure 2D). *E. coli* growth was not increased after 24 h of incubation when stimulated by these vitamins, whereas *E. faecalis* was positively influenced after 24 h of incubation but only when one of the vitamins was added and not with both (*p =* 0.0130; *p* = 0.002) (Figure 2E,F).

### 3.2. In Vivo Findings

#### 3.2.1. Baseline Data

In accordance with the ARRIVE guidelines, baseline characteristics of all experimental groups were analysed at day −1, prior to vitamin supplementation (Table 1). No statistical differences were observed in body weight and plasma citrulline between the different groups.

#### 3.2.2. Vitamin C but Not Vitamin B2 Promotes Body Weight and Food Intake during MTX-Induced Mucositis

**Figure 3:**
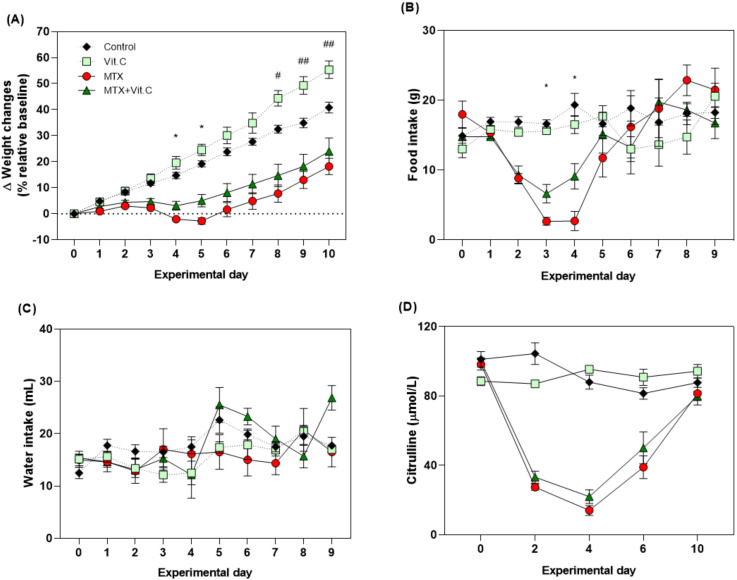
Effects of vitamin C (50 mg/day/rat) on the clinical parameters. (**A**) Bodyweight relative to weight at day 0, which was set at 100%. (**B**) Food intake in grams per day. (**C**) Water intake in ml per day. (**D**) Plasma citrulline level (μmol/L) measured at days 0, 2, 4, 6 and 10 after MTX injection. All data are presented as mean ± SEM. # (*p* < 0.05) and ## (*p* < 0.01) represent the differences between the control group and vitamin C-supplemented rats. * (*p <* 0.05) represent the significant changes between MTX-treated rats supplemented with vitamin C and MTX-treated rats only.

**Figure 4:**
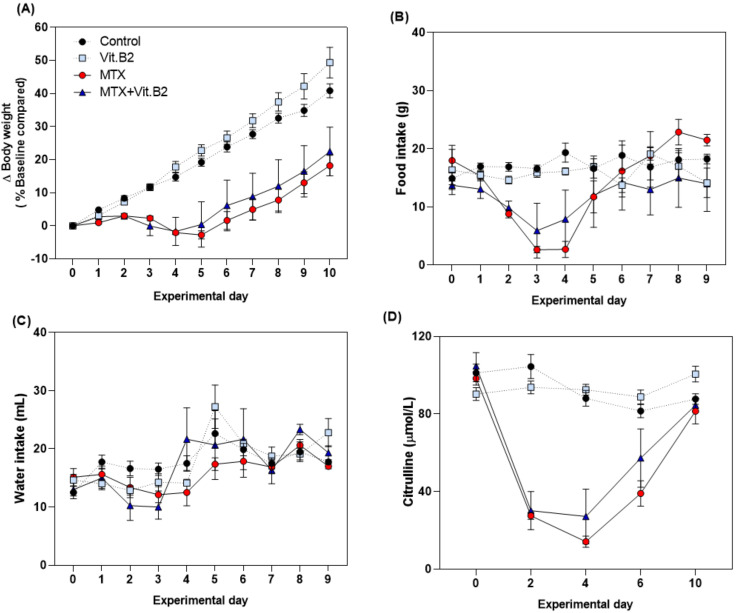
Effects of vitamin B2 (12 mg/day/rat) on the clinical parameters. (**A**) Bodyweight relative to weight at day 0, which was set at 100%. (**B**) Food intake in grams per day. (**C**) Water intake in ml per day. (**D**) Plasma citrulline level (μmol/L) measured at days 0, 2, 4, 6 and 10 after MTX injection. All data are presented as mean ± SEM.

MTX induced a typical clinical phenotype of mucositis which peaked at day 4. Mucositis was characterized by alterations in body weight (−2.056 ± 0.54% vs. 14.74 ± 1.22%, *p* < 0.0001), food intake (2.71 ± 1.38 vs. 19.36 ± 1.64 g, respectively; *p* = 0.0001) and plasma citrulline (14.11 ± 2.86 vs. 88.04 ± 4.02 µmol/L, respectively; *p* < 0.0001), when compared to vehicle control animals (Figure 3 and Figure 4).

Continuous supplementation with 50 mg/day of vitamin C in healthy rats significantly increased body weight compared to controls, as observed at day 10 (55.36 ± 3.35% vs. 40.83 ± 2.09% g, respectively, *p* = 0.018; Figure 3A). However, it did not impact food intake, water intake or plasma citrulline concentrations (Figure 3B–D).

In MTX-treated rats, vitamin C supplementation attenuated weight loss induced by MTX on day 4 (3.09 ± 1.68% vs. −2.056 ± 0.54%, *p =* 0.044) and day 5 (5.066 ± 2.38% vs. −2.77 ± 1.23%, *p* = 0.004; Figure 3A). Food intake was also increased in these animals compared to MTX alone on day 3 (6.63 ± 1.32 vs. 2.66 ± 0.55 g; *p* = 0.015) and day 4 (9.11 ± 1.81 vs. 2.71 ± 1.39 g; *p* = 0.014; Figure 3B). No significant effect was observed in water intake or plasma citrulline (Figure 3C,D). Vitamin B2 did not influence any outcome measures assessed in both healthy and MTX-treated animals (Figure 4). Treatment with vitamins, MTX or the combination of both did not cause diarrhea to the animals. Severe diarrhea, together with weight loss (>20% of the initial weight), was only observed in one rat (MTX+VitB2) that was sacrificed at day 3, leaving a total of N = 3 in this group. Blood and fecal samples from this animal were collected and results included at day 4.

#### 3.2.3. Vitamins Fail to Modulate MTX-Induced Microbial Disruption

**Figure 5:**
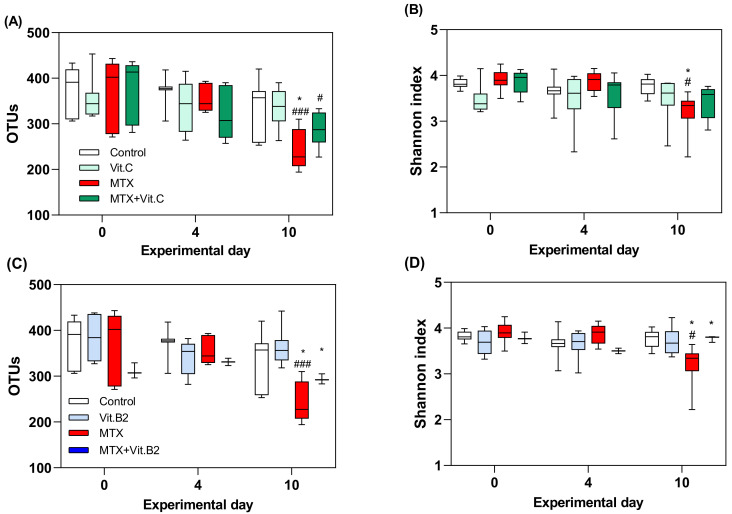
Dynamics of microbial composition defined as alpha-diversity (Shannon) and operational taxonomic unit (OTUs) by day of treatment. OTUs and alpha-diversity from health and MTX-treated rats supplemented with vitamin C represented by (**A**,**B**) and with vitamin B2 by (**C**,**D**). All data are presented as mean ± SEM. # (*p* < 0.05) and ### (*p* < 0.001) represent the significant differences within a group throughout time. * (*p* < 0.05) represent the significant differences between MTX-treated and MTX and vitamin B2-treated rats.

16S rRNA gene analysis was performed in repeated faecal samples collected longitudinally from N = 8 per group, with exception of MTX+VitB2 animals (N = 4), at days 0, 4 and 10. The number of reads of the sequenced samples are listed in Supplementary Materials (Table S2). MTX treatment caused gradual reduction in both alpha diversity (Shannon index) and richness (OTUs) when compared to controls (*p* = 0.0009 and *p* = 0.026, Figure 5). This effect is more pronounced at day 10, where MTX-treated rats present a significant lower OTUs, when compared to the control group (*p* = 0.0333, Figure 5A,C). Continuous supplementation with vitamin C in MTX-treated animals failed to prevent this decrease in microbial richness (*p* = 0.0196, Figure 5A). In contrast, vitamin B2 did not have any influence on microbial diversity or richness in healthy controls. However, it was able to mitigate MTX-induced changes in both alpha diversity and OTUs at day 10 (*p* = 0.0342, *p* = 0.0492; Figure 5C,D).

#### 3.2.4. Vitamin C Supplementation in MTX-Treated Rats Increases *Enterobacteriaceae* Family at Day 4

**Figure 6:**
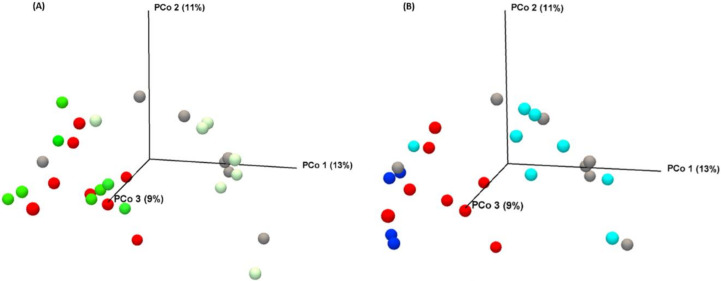
Bacterial profile between different interventions at day 4, after MTX insult. (**A**) PCoA plot of microbial composition (Bray–Curtis dissimilarity on taxomomic level of genera) after vitamin C supplementation. (**B**) PCoA plot after vitamin B2 supplementation.

PERMANOVA analysis (based on Bray–Curtis dissimilarity) confirmed a separation of samples according to the MTX treatment (*p* = 0.00001, Figure 6). However, vitamin C (*p* = 0.13121) and vitamin B2 (*p* = 0.1212) treatment did not cause any significant alteration at day 4 after the MTX treatment. Moreover, no differences in the clustering pattern between vitamin C and vitamin B2 groups were observed (*p* = 0.3408).

**Figure 7:**
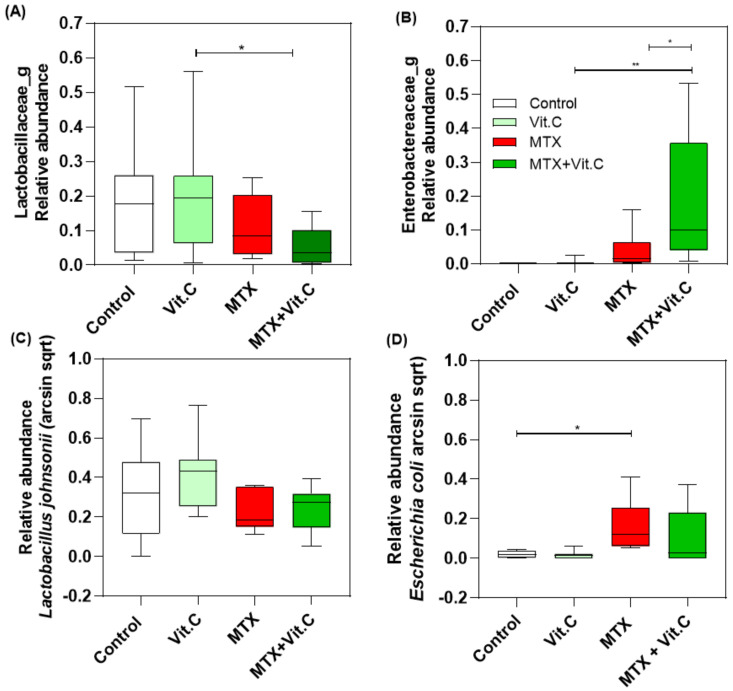
Relative abundance between healthy controls and MTX-treated and MTX and Vitamin C-treated rats at day 4 after MTX insult. Relative abundance of *Lactobaciliaceae* (**A**), *Enterobacteriaceae* (**B**), *Lactobacillus johnsonii* (**C**) and *Escherichia coli* (**D**). Relative abundance of *Lactobacillus johnsonii* and *Escherichia coli* were calculated as arcsin sqrt. * (*p* < 0.05) and ** (*p* < 0.01) represent the significant changes in the relative abundance between the different groups.

Relative abundance (taxonomic level family) showed that the compositional differences at day 4 were driven by losses in *Lactobaciliaceae* with paralleled increases in *Enterobacteriaceae*, which were more profound in MTX+VitC treated rats (*p* = 0.0235; *p* = 0.0296; Figure 7A,B). These alterations were most likely driven by a gradual reduction in *Lactobacillus johnsonii* accompanied by an increase of *Escherichia coli* at day 4 (Figure 7C,D).

#### 3.2.5. Vitamin B2 Induced Changes in the Composition of the Gut Microbiota during Recovery of MTX-Induced Mucositis

**Figure 8:**
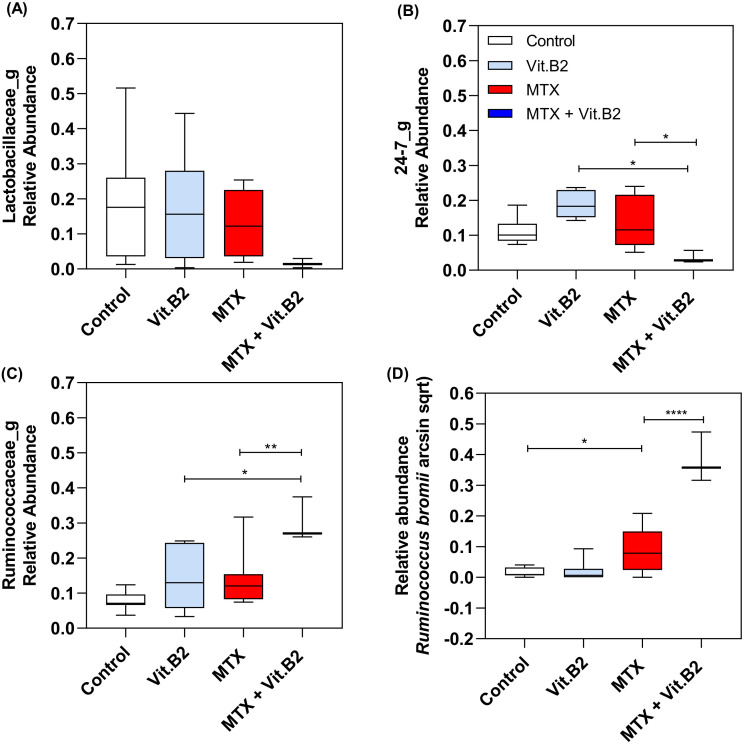
Relative abundance between healthy controls and MTX-treated and MTX and Vitamin B2-treated rats at day 4 after MTX insult. Relative abundance of *Lactobaciliaceae* (**A**), *Muribaculaceae S24-7* (**B**), *Ruminoccoceae* (**C**) and *Ruminococcus bromii* (**D**). Relative abundance of *Lactobacillus johnsonii* and *Escherichia coli* were calculated as arcsin sqrt. * (*p* < 0.05), **(*p* < 0.01) and **** (*p* < 0.0001) represent the significant changes in the relative abundance between the different groups.

Similar to vitamin C, the effects of vitamin B2 were analysed through principal components analysis at day 4. Results showed no significant changes between MTX and MTX+VitB2 groups (*p* = 0.1212; Figure 6). At day 4 post-MTX, Vitamin B2 had influence on the composition of the gut microbiota, with changes in the relative abundance of *Ruminococcaceae* (*p* = 0.0097) and *Muribaculaceae* S24-7 (*p* = 0.0422), when compared to the MTX control group, (Figure 8A–C). Specifically, a significant increase was observed in the relative abundance of *Ruminococcus bromii* in MTX+VitB2, when compared to controls (*p* < 0.0001; Figure 8D).

## 4. Discussion

In this study we demonstrate that prophylactic treatment with vitamin C mitigates the clinical symptoms of MTX-induced mucositis, partially controlling the clinical symptomology of MTX-induced gastrointestinal mucositis such as body weight loss and reduced food intake. In contrast, vitamin B2 was unable to protect against MTX-induced mucositis and in discrete cases, worsened the severity of injury. In both cases, vitamin interventions were unable to modulate the course of MTX-induced mucosal injury, determined by citrulline, despite their influence on microbial composition/integrity demonstrated in vitro and in vivo.

Our understanding of gastrointestinal mucositis has grown enormously over the past few decades, with an increasingly sophisticated pathobiological model describing key events that drive tissue injury and symptoms [28]. Evidence now supports the role of host-microbe interactions in the development of mucositis with changes in the composition of the gut microbiota well documented to coincide with the development of severe mucositis [21,29]. Similarly, augmentation of the microbiota prior to therapy has been shown to modulate the course of mucositis, aligning with new research that points to unique microbial phenotypes that govern mucositis risk [30]. Despite this new insight, our ability to modulate the microbiota in a way that prevents mucositis remains a challenge, with probiotics failing to translate clinically [28,31,32,33]. These underwhelming outcomes suggest that targeting only the microbiota is not sufficient to overcome the intensity of injury caused by chemotherapy and interventions that target multiple disease pathways are of increasing relevance.

Vitamins offer a simple method of targeting microbial and inflammatory mechanisms driving mucositis, with efficacy demonstrated in models of 5-FU mucositis and other benign inflammatory conditions [8,34]. In this study, we focus on two different vitamins: C and B2. Vitamin C is an important radical scavenger that prevents cell membrane oxidation and injury caused by reactive oxygen species. Moreover, this vitamin was also demonstrated to play an important role as anti-cancer modulator [8]. Specifically, vitamin C was shown to induce toxicity in selective oncogene-driven cancers and to slow down tumour growth in animal models [35,36]. We have shown that *F. prausnitzii* is able to use vitamin B2 (or riboflavin) as a redox mediator for extracellular electron transfer [9]. Considering the beneficial properties of *F. prausnitzii* as a butyrate producer, increased amounts of this bacterium may be beneficial in the prevention of mucositis. In a study performed by Xuan et al., 2013 human embryonic lung fibroblasts (Helf cells) were irradiated and then incubated with different concentrations of riboflavin laureate and their proliferative properties were investigated. Results show that when higher concentrations of this compound are added to the cultures, proliferation rates increase significantly suggesting that on a cellular level, riboflavin laureate can enhance cell proliferation in both oral and gastrointestinal mucositis [37]. In a prospective clinical study by von Martels et al., 2019 vitamin B2 supplementation in Crohn’s disease patients resulted in reduced systemic oxidative stress and clinical symptoms, suggesting that this vitamin has the capability to improve inflammatory conditions. However, the mechanism was not via the modulation of the microbiome, since this did not change upon vitamin B2 supplementation.

In the present study, we started by investigating the effects of both vitamins C and B2, alone or in combination, on the in vitro growth of several bacterial strains intrinsically associated with intestinal inflammation [21,24,25,27,38]. Our results show that, contrarily to the reduction that is usually observed during chemotherapy-induced mucositis, vitamin supplementation under oxidative conditions enhanced the growth of *B. coccoides* and *R. intestinalis.* Moreover, no significant differences were observed on the growth of *E. coli*, suggesting that prophylactic treatment with vitamins can restore beneficial bacteria in a dysbiotic situation. These promising results led us to investigate the prophylactic potential of these vitamins in vivo.

Here, we show that vitamin C does not profoundly modulate the gut microbiota during the course of MTX-induced mucositis in rats, however it did influence the abundance of specific microbes including in *Lactobacillaceae*, *Enterobactereaceae*, *L. johnsonii* and *E. coli*. Despite the lack of microbial impact, vitamin C was able to lessen MTX-induced weight loss and promote food intake in peak mucositis, albeit the benefits were mild. This suggests that the documented benefits of vitamin C are most likely due to their immunomodulatory properties, comparable to those reported by Al-asmari et al., 2015 in which daily administration of vitamin C (500 mg/kg) following 5-FU led to a significant reduction of NF-κB activation [8]. The limited benefits of vitamin C also suggest that this approach is not sufficient in mucositis control, or alternatively the schedule of intervention was not sufficient to provide optimal mucosal protection. Studies have suggested a link between the increased amount of *Enterobacteriaceae* in the gut and the development of gut inflammation is several human diseases [39]. The increase in the relative amount of *Enterobacteriaceae* in the MTX+VitC group could therefore explain the lack of a more effective anti-inflammatory response from vitamin C. This finding is in contrast to those by Verghese et al., 2017 who demonstrated that vitamin C is able to prevent, in a dose-dependent manner, the in vitro growth of bacteria such as *E. coli* [40].

Given the severity of bone marrow suppression that MTX causes and risk of blood stream infection, this finding warrants further investigation and emphasizes the need to rigorously assess the safety of such vitamin interventions for mucositis prevention.

Vitamin B2 is a water-soluble vitamin with anti-inflammatory and anti-oxidative properties. Moreover, it has been shown that this vitamin is essential for the growth of butyrate-producing bacteria such as *F. prausnitzii* that uses for extracellular electron shuttling, allowing the bacteria to grow under oxidative conditions [9]. In contrast to vitamin C, vitamin B2 was unable to induce any clinical benefits to MTX-treated rats, and in fact worsened mucositis outcomes in some instances, preventing completion of this experimental group. Its effects on the microbiota were also negligible, despite our in vitro studies supporting beneficial impacts on microbial growth. The disparity between our in vitro and in vivo findings is challenging to dissect, however we hypothesize that the in vitro benefits were likely the result of the anti-oxidant properties of both vitamins, which in turn allowed the bacteria to grow under stress conditions. For example, it is known that vitamin B2 can act as an electron shuttle in anaerobic bacteria, thus facilitating respiration that yields more energy for growth and reduces the environment [9]. Interestingly, in contrast to our results, Levit et al., 2018 showed that administration of a riboflavin-producing strain *Lactobacillus plantarum* to 5-FU-treated mice had a protective effect against chemotherapy-induced mucositis, characterized by lower diarrhea scores, preserved mucosal architecture and maintained of crypt length [41,42]. We hypothesize that, when translated in vivo, vitamin B2 effects are insufficient to overcome the intensity of injury caused by MTX and the hostility of the luminal micro-environment. This is supported by a study by Anandam et al., 2018 who showed that prolonged exposure to pro-inflammatory cytokines such as tumour necrosis factor (TNF) can inhibit intestinal uptake of vitamin B2. This is due to the suppression of the expression of the apical vitamin B2 uptake system- hRFVT-3, a vitamin B2 transporter. In line with these results, we suggest that vitamin B2 did not exert any kind of beneficial effect due to the exacerbated inflammation caused by MTX. According to several studies, TNF is highly expressed during the most severe phase of mucositis. This increased expression might therefore explain the lack of response to this specific dose of vitamin B2 tested.

While there are plausible explanations for the lack of efficacy demonstrated for prophylactic vitamin supplementation, there are several limitations in our study that warrant recognition. To ensure translation of our findings and integration into clinical practice, we employed a short prophylactic regimen, with vitamins administered one day prior to MTX treatment. It is possible that either the amount of vitamins reaching the small intestine was not sufficient to overcome MTX effects or that there was not sufficient time to allow for microbial modulation, with effects only mildly evident at day 10. If indeed the perceived benefits of vitamins are due to their immunomodulatory effects, administration may be optimized to promote restoration of the mucosa in the recovery phase by the recruitment of gut homing reparative lymphocytes (day 7–10). However, these are mere speculations based on previous observations, since we did not access the anti-inflammatory properties of these vitamins and, in the case of vitamin B2 supplementation, the number of animals in the MTX+VitB2 group was considerably low. Therefore, further studies need to be performed in order to investigate the full potential of these vitamins in the set of chemotherapy-induced mucositis.

## 5. Conclusions

In conclusion, the findings of the present study provide critical evidence regarding the challenges in using vitamins to prevent or control MTX-induced mucositis. We have shown the potential of vitamin C in ameliorating mucositis symptoms, yet its effect on the microbiota requires further optimization for improved mucosal protection. Future research on vitamins in mucositis should consider extended prophylaxis to improve magnitude of effect.

## Figures and Tables

**Table 1:** Baseline body weight in each experimental group (mean ± SEM). Animals were randomized according to the body weight and plasma citrulline levels.

Baseline Body Weight in Each Experiments Group		
	Body Weight (g)	Plasma Citrulline (µmol/L)
Control (N = 8)	95.88 ± 0.22	101.16 ± 4.51
Vitamin C (N = 8)	96.34 ± 0.27	88.52 ± 2.53
Vitamin B2 (N = 8)	95.88 ± 0.22	90.29 ± 9.34
MTX (N = 8)	96.39 ± 0.34	98.33 ± 9.59
MTX+VitC (N = 8)	97.40 ± 0.38	100.05 ± 1.99
MTX+VitB2 (N = 4)	97.82 ± 0.88	104.70 ± 13.84

## Data Availability

Not applicable.

## Supplementary Materials

The following are available online at https://www.mdpi.com/2218-273X/11/1/34/s1, Table S1: Sequence of the modified 341F and 806R primers used in this study, Table S2: Number of reads of the sequenced samples at day 4.

## References

[1] Sonis S.T., Elting L.S., Keefe D., Peterson D.E., Schubert M., Hauer-Jensen M., Bekele B.N., Raber-Durlacher J., Donnelly J.P., Rubenstein E.B. (2004). Perspectives on Cancer Therapy-Induced Mucosal Injury: Pathogenesis, Measurement, Epidemiology, and Consequences for Patients. Cancer.

[2] Sonis S.T. (2004). Pathobiology of mucositis. Semin. Oncol. Nurs..

[3] Sonis S.T. (2009). Mucositis: The impact, biology and therapeutic opportunities of oral mucositis. Oral Oncol..

[4] Gubatan J., Moss A.C. (2018). Vitamin D in inflammatory bowel disease: More than just a supplement. Curr. Opin. Gastroenterol..

[5] Wadleigh R.G., Redman R.S., Graham M.L., Krasnow S.H., Anderson A., Cohen M.H. (1992). Vitamin E in the treatment of chemotherapy-induced mucositis. Am. J. Med..

[6] Nejatinamini S., Debenham B.J., Clugston R.D., Mawani A., Parliament M., Wismer W.V., Mazurak V.C. (2018). Poor vitamin status is associated with skeletal muscle loss and mucositis in head and neck cancer patients. Nutrients.

[7] Masri O.A., Chalhoub J.M., Sharara A.I. (2015). Role of vitamins in gastrointestinal diseases. World J. Gastroenterol..

[8] Al-asmari A.K., Khan A.Q., Al-qasim A.M., Al-yousef Y. (2015). Ascorbic acid attenuates antineoplastic drug 5-fluorouracil induced gastrointestinal toxicity in rats by modulating the expression of inflammatory mediators. Toxicol. Rep..

[9] Khan M.T., Browne W.R., van Dijl J.M., Harmsen H.J.M. (2012). How Can *Faecalibacterium prausnitzii* Employ Riboflavin for Extracellular Electron Transfer?. Antioxid. Redox Signal..

[10] Subramanian V.S., Sabui S., Subramenium G.A., Marchant J.S., Said H.M. (2018). Tumor necrosis factor alpha reduces intestinal vitamin C uptake: A role for NF-κB-mediated signaling. Am. J. Physiol. Gastrointest. Liver Physiol..

[11] Powers H.J. (2003). Riboflavin (vitamin B-2) and health. Am. J. Clin. Nutr..

[12] Kondo K., Hiramoto K., Yamate Y., Goto K., Sekijima H., Ooi K. (2019). Ameliorative effect of high-dose Vitamin C administration on dextran sulfate sodium-induced colitis mouse model. Biol. Pharm. Bull..

[13] De Moreno de LeBlanc A., Levit R., de Giori G.S., LeBlanc J.G. (2018). Vitamin Producing Lactic Acid Bacteria as Complementary Treatments for Intestinal Inflammation. Antiinflamm. Antiallergy. Agents Med. Chem..

[14] Levit R., Savoy de Giori G., de Moreno de LeBlanc A., LeBlanc J.G. (2017). Evaluation of the effect of soymilk fermented by a riboflavin-producing Lactobacillus plantarum strain in a murine model of colitis. Benef. Microbes.

[15] Robien K., Schubert M.M., Bruemmer B., Lloid M.E., Potter J.D., Ulrich C.M. (2004). Predictors of oral mucositis in patients receiving hematopoietic cell transplants for chronic myelogenous leukemia. J. Clin. Oncol..

[16] Bartram A.K., Lynch M.D.J., Stearns J.C., Moreno-Hagelsieb G., Neufeld J.D. (2011). Generation of multimillion-sequence 16S rRNA gene libraries from complex microbial communities by assembling paired-end Illumina reads. Appl. Environ. Microbiol..

[17] Yu Z., Morrison M. (2004). Improved extraction of PCR-quality community DNA from digesta and fecal samples. Biotechniques.

[18] Duncan S.H., Hold G.L., Harmsen H.J.M., Stewart C.S., Flint H.J. (2002). Growth requirements and fermentation products of Fusobacterium prausnitzii, and a proposal to reclassify it as Faecalibacterium prausnitzii gen. nov., comb. nov. Int. J. Syst. Evol. Microbiol..

[19] Kuiken N.S.S., Rings E.H.H.M., Alffenaar J.W.C., Havinga R., Jurdzinski A., Groen A.K., Tissing W.J.E. (2017). Tumor Necrosis Factor-Alpha Inhibitor Etanercept Does Not Alter Methotrexate-Induced Gastrointestinal Mucositis in Rats. J. Pediatr. Gastroenterol. Nutr..

[20] Fijlstra M., Tissing W.J.E., Stellaard F., Verkade H.J., Rings E.H.H.M. (2013). Reduced absorption of long-chain fatty acids during methotrexate-induced gastrointestinal mucositis in the rat. Clin. Nutr..

[21] Van Vliet M.J., Harmsen H.J.M., de Bont E.S.J.M., Tissing W.J.E. (2010). The role of intestinal microbiota in the development and severity of chemotherapy-induced mucositis. PLoS Pathog..

[22] Van Vliet M.J., Tissing W.J.E., Rings E.H.H.M., Koetse H.A., Stellaard F., Kamps W.A., De Bont E.S.J.M. (2009). Citrulline as a marker for chemotherapy induced mucosal barrier injury in pediatric patients. Pediatr. Blood Cancer.

[23] Fijlstra M., Rings E.H.H.M., Verkade H.J., van Dijk T.H., Kamps W.A., Tissing W.J.E. (2011). Lactose maldigestion during methotrexate-induced gastrointestinal mucositis in a rat model. Am. J. Physiol. Gastrointest. Liver Physiol..

[24] Venegas D.P., De La Fuente M.K., Landskron G., González M.J., Quera R., Dijkstra G., Harmsen H.J.M., Faber K.N., Hermoso M.A. (2019). Short chain fatty acids (SCFAs)mediated gut epithelial and immune regulation and its relevance for inflammatory bowel diseases. Front. Immunol..

[25] Jenq R.R., Taur Y., Devlin S.M., Ponce D.M., Goldberg J.D., Ahr K.F., Littmann E.R., Ling L., Gobourne A.C., Miller L.C. (2015). Intestinal Blautia Is Associated with Reduced Death from Graft-versus-Host Disease. Biol. Blood Marrow Transplant..

[26] Geirnaert A., Calatayud M., Grootaert C., Laukens D., Devriese S., Smagghe G., De Vos M., Boon N., Van De Wiele T. (2017). Butyrate-producing bacteria supplemented in vitro to Crohn’s disease patient microbiota increased butyrate production and enhanced intestinal epithelial barrier integrity. Sci. Rep..

[27] Touchefeu Y., Montassier E., Nieman K., Gastinne T., Potel G., Bruley Des Varannes S., Le Vacon F., De La Cochetière M.F. (2014). Systematic review: The role of the gut microbiota in chemotherapy- or radiation-induced gastrointestinal mucositis - Current evidence and potential clinical applications. Aliment. Pharmacol. Ther..

[28] Bowen J., Al-Dasooqi N., Bossi P., Wardill H., Van Sebille Y., Al-Azri A., Bateman E., Correa M.E., Raber-Durlacher J., Kandwal A. (2019). The pathogenesis of mucositis: Updated perspectives and emerging targets. Support. Care Cancer.

[29] Louise Pouncey A., James Scott A., Leslie Alexander J., Marchesi J., Kinross J. (2018). Gut microbiota, chemotherapy and the host: The influence of the gut microbiota on cancer treatment. Ecancermedicalscience.

[30] Alexander J.L., Wilson I.D., Teare J., Marchesi J.R., Nicholson J.K., Kinross J.M. (2017). Gut microbiota modulation of chemotherapy efficacy and toxicity. Nat. Rev. Gastroenterol. Hepatol..

[31] Wardill H.R., Tissing W.J.E. (2017). Determining risk of severe gastrointestinal toxicity based on pretreatment gut microbial community in patients receiving cancer treatment. Curr. Opin. Support. Palliat. Care.

[32] Cereda E., Caraccia M., Caccialanza R. (2018). Probiotics and mucositis. Curr. Opin. Clin. Nutr. Metab. Care.

[33] Prisciandaro L.D., Geier M.S., Butler R.N., Cummins A.G., Howarth G.S. (2011). Evidence supporting the use of probiotics for the prevention and treatment of chemotherapy-induced intestinal mucositis. Crit. Rev. Food Sci. Nutr..

[34] Meeker S., Seamons A., Maggio-Price L., Paik J. (2016). Protective links between Vitamin D, inflammatory bowel disease and colon cancer. World J. Gastroenterol..

[35] Vissers M.C.M., Das A.B. (2018). Potential mechanisms of action for vitamin C in cancer: Reviewing the evidence. Front. Physiol..

[36] Van Der Reest J., Gottlieb E. (2016). Anti-cancer effects of Vitamin C revisited. Cell Res..

[37] Xuan Z., An Y., Yang D., Wang S., Xu Q., Yuan S. (2013). Exploration of the protection of riboflavin laurate on oral mucositis induced by chemotherapy or radiotherapy at the cellular level: What is the leading contributor?. Int. J. Mol. Sci..

[38] Li H.-L., Lu L., Wang X.-S., Qin L.-Y., Wang P., Qiu S.-P., Wu H., Huang F., Zhang B.-B., Shi H.-L. (2017). Alteration of Gut Microbiota and Inflammatory Cytokine/Chemokine Profiles in 5-Fluorouracil Induced Intestinal Mucositis. Front. Cell. Infect. Microbiol..

[39] Zeng M.Y., Inohara N., Nuñez G. (2017). Mechanisms of inflammation-driven bacterial dysbiosis in the gut. Mucosal Immunol..

[40] Verghese R., Mathew S., David A. (2017). Antimicrobial activity of Vitamin C demonstrated on uropathogenic Escherichia coli and Klebsiella pneumoniae. J. Curr. Res. Sci. Med..

[41] Levit R., Savoy de Giori G., de Moreno de LeBlanc A., LeBlanc J.G. (2018). Protective effect of the riboflavin-overproducing strain Lactobacillus plantarum CRL2130 on intestinal mucositis in mice. Nutrition.

[42] De Moreno de LeBlanc A., Perdigón G. (2010). The application of probiotic fermented milks in cancer and intestinal inflammation. Proc. Nutr. Soc..

