# Supplementary material for: Prophylactic Treatment with Vitamins C and B2 for Methotrexate-Induced Gastrointestinal Mucositis
Source: Biomolecules. 2020 Dec 29;11(1):34. doi: 10.3390/biom11010034 (PMC7823339; doi:10.3390/biom11010034)
Supplement: Supplementary file 1 [file biomolecules-11-00034-s001.pdf]

Supplementary Materials

Table S1. Sequence of the modified 341F and 806R primers used in this study

V3_F_modified	aatgatacggcgaccaccgagatct	acactctttccctacacgacgctcttccgatct		NNNNCCTACGGGAGGCAGCAG
V4_1R	caagcagaagacggcatacgagat	**ATCACG**	gtgactggagttcagacgtgtgctcttccgatct	GGACTACHVGGGTWTCTAAT
V4_2R	caagcagaagacggcatacgagat	**CGATGT**	gtgactggagttcagacgtgtgctcttccgatct	GGACTACHVGGGTWTCTAAT
V4_3R	caagcagaagacggcatacgagat	**TTAGGC**	gtgactggagttcagacgtgtgctcttccgatct	GGACTACHVGGGTWTCTAAT
V4_4R	caagcagaagacggcatacgagat	**TGACCA**	gtgactggagttcagacgtgtgctcttccgatct	GGACTACHVGGGTWTCTAAT
V4_5R	caagcagaagacggcatacgagat	**ACAGTG**	gtgactggagttcagacgtgtgctcttccgatct	GGACTACHVGGGTWTCTAAT
V4_6R	caagcagaagacggcatacgagat	**GCCAAT**	gtgactggagttcagacgtgtgctcttccgatct	GGACTACHVGGGTWTCTAAT
V4_7R	caagcagaagacggcatacgagat	**CAGATC**	gtgactggagttcagacgtgtgctcttccgatct	GGACTACHVGGGTWTCTAAT
V4_8R	caagcagaagacggcatacgagat	**ACTTGA**	gtgactggagttcagacgtgtgctcttccgatct	GGACTACHVGGGTWTCTAAT
V4_9R	caagcagaagacggcatacgagat	**GATCAG**	gtgactggagttcagacgtgtgctcttccgatct	GGACTACHVGGGTWTCTAAT
V4_10R	caagcagaagacggcatacgagat	**TAGCTT**	gtgactggagttcagacgtgtgctcttccgatct	GGACTACHVGGGTWTCTAAT
V4_11R	caagcagaagacggcatacgagat	**GGCTAC**	gtgactggagttcagacgtgtgctcttccgatct	GGACTACHVGGGTWTCTAAT
V4_12R	caagcagaagacggcatacgagat	**CTTGTA**	gtgactggagttcagacgtgtgctcttccgatct	GGACTACHVGGGTWTCTAAT
V4_13R	caagcagaagacggcatacgagat	**AGTACG**	gtgactggagttcagacgtgtgctcttccgatct	GGACTACHVGGGTWTCTAAT
V4_14R	caagcagaagacggcatacgagat	**TCAGTC**	gtgactggagttcagacgtgtgctcttccgatct	GGACTACHVGGGTWTCTAAT
V4_15R	caagcagaagacggcatacgagat	**TTGAGC**	gtgactggagttcagacgtgtgctcttccgatct	GGACTACHVGGGTWTCTAAT
V4_16R	caagcagaagacggcatacgagat	**AAGCGA**	gtgactggagttcagacgtgtgctcttccgatct	GGACTACHVGGGTWTCTAAT
V4_17R	caagcagaagacggcatacgagat	**TCCTCA**	gtgactggagttcagacgtgtgctcttccgatct	GGACTACHVGGGTWTCTAAT
V4_18R	caagcagaagacggcatacgagat	**GGTTGT**	gtgactggagttcagacgtgtgctcttccgatct	GGACTACHVGGGTWTCTAAT
V4_19R	caagcagaagacggcatacgagat	**TGAGGT**	gtgactggagttcagacgtgtgctcttccgatct	GGACTACHVGGGTWTCTAAT
V4_20R	caagcagaagacggcatacgagat	**TACCGT**	gtgactggagttcagacgtgtgctcttccgatct	GGACTACHVGGGTWTCTAAT
V4_21R	caagcagaagacggcatacgagat	**CCAACT**	gtgactggagttcagacgtgtgctcttccgatct	GGACTACHVGGGTWTCTAAT
V4_22R	caagcagaagacggcatacgagat	**AGAGAG**	gtgactggagttcagacgtgtgctcttccgatct	GGACTACHVGGGTWTCTAAT
V4_23R	caagcagaagacggcatacgagat	**CACTTG**	gtgactggagttcagacgtgtgctcttccgatct	GGACTACHVGGGTWTCTAAT
V4_24R	caagcagaagacggcatacgagat	**TCAAGG**	gtgactggagttcagacgtgtgctcttccgatct	GGACTACHVGGGTWTCTAAT
V4_25R	caagcagaagacggcatacgagat	**AGTGGT**	gtgactggagttcagacgtgtgctcttccgatct	GGACTACHVGGGTWTCTAAT
V4_26R	caagcagaagacggcatacgagat	**GACACT**	gtgactggagttcagacgtgtgctcttccgatct	GGACTACHVGGGTWTCTAAT
V4_27R	caagcagaagacggcatacgagat	**CCTTCT**	gtgactggagttcagacgtgtgctcttccgatct	GGACTACHVGGGTWTCTAAT
V4_28R	caagcagaagacggcatacgagat	**GGATAA**	gtgactggagttcagacgtgtgctcttccgatct	GGACTACHVGGGTWTCTAAT
V4_29R	caagcagaagacggcatacgagat	**CCTTAA**	gtgactggagttcagacgtgtgctcttccgatct	GGACTACHVGGGTWTCTAAT
V4_30R	caagcagaagacggcatacgagat	**CAAGAA**	gtgactggagttcagacgtgtgctcttccgatct	GGACTACHVGGGTWTCTAAT
V4_31R	caagcagaagacggcatacgagat	**GTTGAA**	gtgactggagttcagacgtgtgctcttccgatct	GGACTACHVGGGTWTCTAAT
V4_32R	caagcagaagacggcatacgagat	**TCACAA**	gtgactggagttcagacgtgtgctcttccgatct	GGACTACHVGGGTWTCTAAT
V4_33R	caagcagaagacggcatacgagat	**AGTCAA**	gtgactggagttcagacgtgtgctcttccgatct	GGACTACHVGGGTWTCTAAT
V4_34R	caagcagaagacggcatacgagat	**CGAATA**	gtgactggagttcagacgtgtgctcttccgatct	GGACTACHVGGGTWTCTAAT
V4_35R	caagcagaagacggcatacgagat	**GCTATA**	gtgactggagttcagacgtgtgctcttccgatct	GGACTACHVGGGTWTCTAAT

V4_36R	caagcagaagacggcatacgagat	**GAGTTA**	gtgactggagttcagacgtgtgctcttccgatct	GGACTACHVGGGTWTCTAAT
V4_37R	caagcagaagacggcatacgagat	**TTGGTA**	gtgactggagttcagacgtgtgctcttccgatct	GGACTACHVGGGTWTCTAAT
V4_38R	caagcagaagacggcatacgagat	**AACGTA**	gtgactggagttcagacgtgtgctcttccgatct	GGACTACHVGGGTWTCTAAT
V4_39R	caagcagaagacggcatacgagat	**GTACTA**	gtgactggagttcagacgtgtgctcttccgatct	GGACTACHVGGGTWTCTAAT
V4_40R	caagcagaagacggcatacgagat	**CATCTA**	gtgactggagttcagacgtgtgctcttccgatct	GGACTACHVGGGTWTCTAAT
V4_41R	caagcagaagacggcatacgagat	**TGTAGA**	gtgactggagttcagacgtgtgctcttccgatct	GGACTACHVGGGTWTCTAAT
V4_42R	caagcagaagacggcatacgagat	**ATCAGA**	gtgactggagttcagacgtgtgctcttccgatct	GGACTACHVGGGTWTCTAAT
V4_43R	caagcagaagacggcatacgagat	**ACATGA**	gtgactggagttcagacgtgtgctcttccgatct	GGACTACHVGGGTWTCTAAT
V4_44R	caagcagaagacggcatacgagat	**TAGACA**	gtgactggagttcagacgtgtgctcttccgatct	GGACTACHVGGGTWTCTAAT
V4_45R	caagcagaagacggcatacgagat	**GAGAAT**	gtgactggagttcagacgtgtgctcttccgatct	GGACTACHVGGGTWTCTAAT
V4_46R	caagcagaagacggcatacgagat	**CTCAAT**	gtgactggagttcagacgtgtgctcttccgatct	GGACTACHVGGGTWTCTAAT
V4_47R	caagcagaagacggcatacgagat	**AGGTAT**	gtgactggagttcagacgtgtgctcttccgatct	GGACTACHVGGGTWTCTAAT
V4_48R	caagcagaagacggcatacgagat	**TTGCAT**	gtgactggagttcagacgtgtgctcttccgatct	GGACTACHVGGGTWTCTAAT
V4_49R	caagcagaagacggcatacgagat	**TGGATT**	gtgactggagttcagacgtgtgctcttccgatct	GGACTACHVGGGTWTCTAAT
V4_50R	caagcagaagacggcatacgagat	**ACCATT**	gtgactggagttcagacgtgtgctcttccgatct	GGACTACHVGGGTWTCTAAT
V4_51R	caagcagaagacggcatacgagat	**CTAGTT**	gtgactggagttcagacgtgtgctcttccgatct	GGACTACHVGGGTWTCTAAT
V4_52R	caagcagaagacggcatacgagat	**AGTGTT**	gtgactggagttcagacgtgtgctcttccgatct	GGACTACHVGGGTWTCTAAT
V4_53R	caagcagaagacggcatacgagat	**TCTCTT**	gtgactggagttcagacgtgtgctcttccgatct	GGACTACHVGGGTWTCTAAT
V4_54R	caagcagaagacggcatacgagat	**GTAAGT**	gtgactggagttcagacgtgtgctcttccgatct	GGACTACHVGGGTWTCTAAT
V4_55R	caagcagaagacggcatacgagat	**CAATGT**	gtgactggagttcagacgtgtgctcttccgatct	GGACTACHVGGGTWTCTAAT
V4_56R	caagcagaagacggcatacgagat	**ATTCGT**	gtgactggagttcagacgtgtgctcttccgatct	GGACTACHVGGGTWTCTAAT
V4_57R	caagcagaagacggcatacgagat	**ATGACT**	gtgactggagttcagacgtgtgctcttccgatct	GGACTACHVGGGTWTCTAAT
V4_58R	caagcagaagacggcatacgagat	**ACTTCT**	gtgactggagttcagacgtgtgctcttccgatct	GGACTACHVGGGTWTCTAAT
V4_59R	caagcagaagacggcatacgagat	**CATAAG**	gtgactggagttcagacgtgtgctcttccgatct	GGACTACHVGGGTWTCTAAT
V4_60R	caagcagaagacggcatacgagat	**TTCTAG**	gtgactggagttcagacgtgtgctcttccgatct	GGACTACHVGGGTWTCTAAT
V4_61R	caagcagaagacggcatacgagat	**AAGATG**	gtgactggagttcagacgtgtgctcttccgatct	GGACTACHVGGGTWTCTAAT
V4_62R	caagcagaagacggcatacgagat	**TATGTG**	gtgactggagttcagacgtgtgctcttccgatct	GGACTACHVGGGTWTCTAAT
V4_63R	caagcagaagacggcatacgagat	**AATTGG**	gtgactggagttcagacgtgtgctcttccgatct	GGACTACHVGGGTWTCTAAT
V4_64R	caagcagaagacggcatacgagat	**TAATCG**	gtgactggagttcagacgtgtgctcttccgatct	GGACTACHVGGGTWTCTAAT
V4_65R	caagcagaagacggcatacgagat	**ACTAAC**	gtgactggagttcagacgtgtgctcttccgatct	GGACTACHVGGGTWTCTAAT
V4_66R	caagcagaagacggcatacgagat	**TGTTAC**	gtgactggagttcagacgtgtgctcttccgatct	GGACTACHVGGGTWTCTAAT
V4_67R	caagcagaagacggcatacgagat	**ATACAC**	gtgactggagttcagacgtgtgctcttccgatct	GGACTACHVGGGTWTCTAAT
V4_68R	caagcagaagacggcatacgagat	**CTTATC**	gtgactggagttcagacgtgtgctcttccgatct	GGACTACHVGGGTWTCTAAT
V4_69R	caagcagaagacggcatacgagat	**AGATTC**	gtgactggagttcagacgtgtgctcttccgatct	GGACTACHVGGGTWTCTAAT
V4_70R	caagcagaagacggcatacgagat	**ACGGAA**	gtgactggagttcagacgtgtgctcttccgatct	GGACTACHVGGGTWTCTAAT
V4_71R	caagcagaagacggcatacgagat	**TGCGAA**	gtgactggagttcagacgtgtgctcttccgatct	GGACTACHVGGGTWTCTAAT
V4_72R	caagcagaagacggcatacgagat	**GACCAA**	gtgactggagttcagacgtgtgctcttccgatct	GGACTACHVGGGTWTCTAAT
V4_73R	caagcagaagacggcatacgagat	**CTGTCA**	gtgactggagttcagacgtgtgctcttccgatct	GGACTACHVGGGTWTCTAAT
V4_74R	caagcagaagacggcatacgagat	**GCAGAT**	gtgactggagttcagacgtgtgctcttccgatct	GGACTACHVGGGTWTCTAAT

V4_75R	caagcagaagacggcatacgagat	**TCGTGT**	gtgactggagttcagacgtgtgctcttccgatct	GGACTACHVGGGTWTCTAAT
V4_76R	caagcagaagacggcatacgagat	**GAACCT**	gtgactggagttcagacgtgtgctcttccgatct	GGACTACHVGGGTWTCTAAT
V4_77R	caagcagaagacggcatacgagat	**GTCATG**	gtgactggagttcagacgtgtgctcttccgatct	GGACTACHVGGGTWTCTAAT
V4_78R	caagcagaagacggcatacgagat	**GATAGC**	gtgactggagttcagacgtgtgctcttccgatct	GGACTACHVGGGTWTCTAAT
V4_79R	caagcagaagacggcatacgagat	**AAGTCC**	gtgactggagttcagacgtgtgctcttccgatct	GGACTACHVGGGTWTCTAAT
V4_80R	caagcagaagacggcatacgagat	**ATTGCC**	gtgactggagttcagacgtgtgctcttccgatct	GGACTACHVGGGTWTCTAAT
V4_81R	caagcagaagacggcatacgagat	**CCGAGA**	gtgactggagttcagacgtgtgctcttccgatct	GGACTACHVGGGTWTCTAAT
V4_82R	caagcagaagacggcatacgagat	**CGCTGA**	gtgactggagttcagacgtgtgctcttccgatct	GGACTACHVGGGTWTCTAAT
V4_83R	caagcagaagacggcatacgagat	**GGCACA**	gtgactggagttcagacgtgtgctcttccgatct	GGACTACHVGGGTWTCTAAT
V4_84R	caagcagaagacggcatacgagat	**CGTGCA**	gtgactggagttcagacgtgtgctcttccgatct	GGACTACHVGGGTWTCTAAT
V4_85R	caagcagaagacggcatacgagat	**GGCCTT**	gtgactggagttcagacgtgtgctcttccgatct	GGACTACHVGGGTWTCTAAT
V4_86R	caagcagaagacggcatacgagat	**CCTGGT**	gtgactggagttcagacgtgtgctcttccgatct	GGACTACHVGGGTWTCTAAT
V4_87R	caagcagaagacggcatacgagat	**CAGGCT**	gtgactggagttcagacgtgtgctcttccgatct	GGACTACHVGGGTWTCTAAT
V4_88R	caagcagaagacggcatacgagat	**GTCGCT**	gtgactggagttcagacgtgtgctcttccgatct	GGACTACHVGGGTWTCTAAT
V4_89R	caagcagaagacggcatacgagat	**GCGTAG**	gtgactggagttcagacgtgtgctcttccgatct	GGACTACHVGGGTWTCTAAT
V4_90R	caagcagaagacggcatacgagat	**CTGGAG**	gtgactggagttcagacgtgtgctcttccgatct	GGACTACHVGGGTWTCTAAT
V4_91R	caagcagaagacggcatacgagat	**CTACGG**	gtgactggagttcagacgtgtgctcttccgatct	GGACTACHVGGGTWTCTAAT
V4_92R	caagcagaagacggcatacgagat	**ACACCG**	gtgactggagttcagacgtgtgctcttccgatct	GGACTACHVGGGTWTCTAAT
V4_93R	caagcagaagacggcatacgagat	**GTTCCG**	gtgactggagttcagacgtgtgctcttccgatct	GGACTACHVGGGTWTCTAAT
V4_94R	caagcagaagacggcatacgagat	**CAGCAC**	gtgactggagttcagacgtgtgctcttccgatct	GGACTACHVGGGTWTCTAAT
V4_95R	caagcagaagacggcatacgagat	**CCGTTC**	gtgactggagttcagacgtgtgctcttccgatct	GGACTACHVGGGTWTCTAAT
V4_96R	caagcagaagacggcatacgagat	**GCATCC**	gtgactggagttcagacgtgtgctcttccgatct	GGACTACHVGGGTWTCTAAT
V4_97R	caagcagaagacggcatacgagat	**TACGCC**	gtgactggagttcagacgtgtgctcttccgatct	GGACTACHVGGGTWTCTAAT
V4_98R	caagcagaagacggcatacgagat	**CCGAGT**	gtgactggagttcagacgtgtgctcttccgatct	GGACTACHVGGGTWTCTAAT
V4_99R	caagcagaagacggcatacgagat	**CGAGCT**	gtgactggagttcagacgtgtgctcttccgatct	GGACTACHVGGGTWTCTAAT
V4_100R	caagcagaagacggcatacgagat	**GCGACT**	gtgactggagttcagacgtgtgctcttccgatct	GGACTACHVGGGTWTCTAAT
V4_101R	caagcagaagacggcatacgagat	**CAGACT**	gtgactggagttcagacgtgtgctcttccgatct	GGACTACHVGGGTWTCTAAT
V4_102R	caagcagaagacggcatacgagat	**CCGATG**	gtgactggagttcagacgtgtgctcttccgatct	GGACTACHVGGGTWTCTAAT
V4_103R	caagcagaagacggcatacgagat	**CTGCAG**	gtgactggagttcagacgtgtgctcttccgatct	GGACTACHVGGGTWTCTAAT
V4_104R	caagcagaagacggcatacgagat	**CAGGTC**	gtgactggagttcagacgtgtgctcttccgatct	GGACTACHVGGGTWTCTAAT
V4_105R	caagcagaagacggcatacgagat	**GACGTC**	gtgactggagttcagacgtgtgctcttccgatct	GGACTACHVGGGTWTCTAAT
V4_106R	caagcagaagacggcatacgagat	**GCAGTC**	gtgactggagttcagacgtgtgctcttccgatct	GGACTACHVGGGTWTCTAAT
V4_107R	caagcagaagacggcatacgagat	**ACGATC**	gtgactggagttcagacgtgtgctcttccgatct	GGACTACHVGGGTWTCTAAT
V4_108R	caagcagaagacggcatacgagat	**GGCATC**	gtgactggagttcagacgtgtgctcttccgatct	GGACTACHVGGGTWTCTAAT
V4_109R	caagcagaagacggcatacgagat	**GGTACC**	gtgactggagttcagacgtgtgctcttccgatct	GGACTACHVGGGTWTCTAAT
V4_110R	caagcagaagacggcatacgagat	**TGGACC**	gtgactggagttcagacgtgtgctcttccgatct	GGACTACHVGGGTWTCTAAT
V4_111R	caagcagaagacggcatacgagat	**GCGTAC**	gtgactggagttcagacgtgtgctcttccgatct	GGACTACHVGGGTWTCTAAT
V4_112R	caagcagaagacggcatacgagat	**GTGCAC**	gtgactggagttcagacgtgtgctcttccgatct	GGACTACHVGGGTWTCTAAT
V4_113R	caagcagaagacggcatacgagat	**TGGCAC**	gtgactggagttcagacgtgtgctcttccgatct	GGACTACHVGGGTWTCTAAT

V4_114R	caagcagaagacggcatacgagat	**CCGATA**	gtgactggagttcagacgtgtgctcttccgatct	GGACTACHVGGGTWTCTAAT
V4_115R	caagcagaagacggcatacgagat	**CGTCGA**	gtgactggagttcagacgtgtgctcttccgatct	GGACTACHVGGGTWTCTAAT
V4_116R	caagcagaagacggcatacgagat	**GTCCGA**	gtgactggagttcagacgtgtgctcttccgatct	GGACTACHVGGGTWTCTAAT
V4_117R	caagcagaagacggcatacgagat	**GCTGCA**	gtgactggagttcagacgtgtgctcttccgatct	GGACTACHVGGGTWTCTAAT
V4_118R	caagcagaagacggcatacgagat	**CTGGCA**	gtgactggagttcagacgtgtgctcttccgatct	GGACTACHVGGGTWTCTAAT
V4_119R	caagcagaagacggcatacgagat	**GGTCCA**	gtgactggagttcagacgtgtgctcttccgatct	GGACTACHVGGGTWTCTAAT
V4_120R	caagcagaagacggcatacgagat	**GTGCCA**	gtgactggagttcagacgtgtgctcttccgatct	GGACTACHVGGGTWTCTAAT

Lowercase letters denote adapter sequences necessary for binding to the flow cell, underlined lowercase are binding sites for the Illumina sequencing primers, bold uppercase highlight the index sequences (all the indexes were obtained from Illumina) and regular uppercase are the V3 region forward primer 341F and the V4 region reverse primers 806R. The inclusion of four maximally degenerated bases ("NNNN") maximizes diversity during the first four bases of the run.

Table S2. Number of reads of the sequenced samples at day 4.

Sample number	#animal	Treatment	#reads
1	1	PBS	24995
2	2	PBS	24991
3	3	PBS	24990
4	4	PBS	24945
5	5	PBS	24842
6	6	PBS	24614
7	7	PBS	24837
8	9	VitC	24995
9	10	VitC	24947
10	11	VitC	24719
11	12	VitC	24305
12	13	VitC	24773
13	14	VitC	24784
14	15	VitC	24814
15	16	VitC	24538
16	17	VitB2	24994
17	18	VitB2	24696
18	19	VitB2	24731
19	20	VitB2	24716
20	21	VitB2	24643
21	22	VitB2	24727

22	23	VitB2	24453
23	24	VitB2	24835
24	25	MTX	24508
25	26	MTX	24794
26	27	MTX	24829
27	28	MTX	24830
28	29	MTX	24993
29	30	MTX	24875
30	31	MTX	24986
31	32	MTX	24123
32	33	MTX+VitC	24851
33	34	MTX+VitC	24843
34	35	MTX+VitC	24857
35	36	MTX+VitC	24907
36	37	MTX+VitC	24286
37	38	MTX+VitC	24792
38	39	MTX+VitC	24133
39	40	MTX+VitC	24685
40	41	MTX+VitB2	24984
41	42	MTX+VitB2	24995
42	43	MTX+VitB2	24998
43	44	MTX+VitB2**	24995

*Sample from one animal (PBS group) could not be collected. Therefore only 7 samples were included at this timepoint.
**Sample collected at day 3 (sacrifice).
